# Supplementary material for: Evaluation of the virtual care experience for persons in prospective cohorts with HIV during the COVID pandemic
Source: PLOS Digit Health. 2025 May 12;4(5):e0000857. doi: 10.1371/journal.pdig.0000857 (PMC12068565; doi:10.1371/journal.pdig.0000857)
Supplement: Attachment — Submitted filename: Review_PDIG-D-25-00063.pdf [file pdig.0000857.s002.pdf]

Review_PDIG-D-25-00063

No.	Comment	Part	Action
1	• The title is long.<br>• I suggest simplifying it	Title	
2	**Background/Objectives**<br>• Although there is a hypothesis, the objective is not clearly stated in the abstract. Please add a clear aim for this investigation.<br>• Also please refine the hypothesis to match the results or add briefly the impact of the three mentioned factors (comorbidity, coinfection, addiction or mental health issues) on the virtual care findings.<br>**Methods**<br>• Please specify if the 18-item self- administered questionnaires were validated? Also, if reliability testing was performed?<br>• Also specify the data collection method for laboratory results.<br>• The terms: “COCO study” and “CD4 counts” might not be familiar for the general reader. Consider defining them.<br>• Add a sentence specifying the statistical methods and software used.<br>**Results/ Key Findings**<br>• Please add supporting statistics for the statements: “Access to and difficulty with technology was not a barrier to our older participants” and “Those with a detectable HIV viral load were more likely to engage in virtual care”.<br>**Conclusions**<br>• Please avoid a generic statement in conclusion and kindly specify what recommendations study can suggest based on the specific findings.<br>• Please elaborate on how can virtual health policymakers or health organizations address the identified elements in the study. Example may include a targeted outreach programs to ensure all eligible patients are aware of virtual care keeping in mind the patients’ preferences.	Abstract	
3	• Please provide reference(s)/citation(s) for the statement: “Many physician offices and hospital clinics struggled with prioritizing patients for in person assessment”. Also was this specific to HIV care or healthcare in general?<br>• Please provide reference(s)/citation(s) for the statement: “As the population of persons living with HIV in Canada and elsewhere are more economically disadvantaged, there was concern about access to virtual care”<br>• Please add stats on economic disparities among people living with HIV in Canada.<br>• Please clarify how privacy concerns and HIV prevention measures are important in shared living spaces.<br>• Please provide reference(s)/citation(s) for: “With fear of contracting COVID-19 and the disruption of laboratory services, there was concern regarding delays in regular	Introduction	

<table>
<tr><td></td><td>laboratory testing with negative consequences including missed adverse drug effects, failure to detect and intervene on HIV viral failure or non-adherence and undetected sexually transmitted infections”.
• Please provide the full name of all abbreviations</td><td></td><td></td></tr>
<tr><td>4</td><td>• Please provide the methods section after the introduction. This will help ensure clarity and smooth flow of the study.
• Please specify he study design in clear statement.
• Mention the exact period when participants completed the questionnaire. As mentioned in the abstract, data collection occurred between February 2021 and March 2023.
• Please clarify how participants were recruited?
• The Virtual Care Questionnaire was used. Any content validation?
Any pilot testing? This is required to ensure participant comprehension before full implementation. This is important given the study population, including older adults and individuals with potential mental health or substance use challenges.</td><td>Methodology</td><td></td></tr>
<tr><td>5</td><td>• Results
• “454 participants completed the questionnaire”: Please provide more explanation for the recruitment process and criteria for eligible participants.
• Please more details on the main reasons for lack of virtual care offerings.</td><td>Results</td><td></td></tr>
<tr><td></td><td>• Address how study findings are relevant to global healthcare systems in similar settings.
• Expand discussion on the impact of COVID-19 pandemic as the study was covering the pandemic.
• Discuss how do the % of satisfaction results compare to expectations/targets/benchmarks or previous studies?
• Based on the study findings, please explain in more details the reasons for higher healthcare engagement among older adults.
• Please suggest what types of outreach programs are most effective in enhancing virtual care access and acceptance. Also compare in more details virtual care use vs. in-person care during the study period. You can provide a comparison table
<table>
<tr><th>Factor</th><th>Virtual Care</th><th>In-Person Care</th></tr>
<tr><td>Utilization rate</td><td></td><td></td></tr>
<tr><td>Satisfaction</td><td></td><td></td></tr>
<tr><td>Perceived advantages</td><td></td><td></td></tr>
<tr><td>Main barriers</td><td></td><td></td></tr>
<tr><td>Impact on laboratory testing</td><td></td><td></td></tr>
<tr><td>Medication refills</td><td></td><td></td></tr>
<tr><td>Access to multidisciplinary care</td><td></td><td></td></tr>
</table>
</td><td>Discussion</td><td></td></tr>
</table>

	• Please add study limitations:<br>1. The study participants were actively enrolled in ongoing cohort studies and they may not reflect the broader HIV population.<br>2. The bias, which could influence responses regarding satisfaction and engagement in care.<br>3. Findings may not be fully applicable to other healthcare settings outside of Canada.	Limitations	
	• Please suggest some directions for future studies based on your study limitations and unresolved issues.<br>• Also please consider the impact of changes in policies in Canada (if any).<br>• Future studies can explore how do telehealth reimbursement models affect provider participation and patient access to virtual HIV care?	Recommendation for future studies	
	• Please suggest specific strategies for improving in virtual care vulnerable communities.<br>• Please add in brief how the study results can inform policymakers or healthcare planning teams<br>• Add how can virtual care platforms better integrate multidisciplinary teams to improve holistic HIV care.	Conclusion	
	• Please add citation/references whenever indicated.	References	
