# Supplementary material for: Evaluation of the virtual care experience for persons in prospective cohorts with HIV during the COVID pandemic
Source: PLOS Digit Health. 2025 May 12;4(5):e0000857. doi: 10.1371/journal.pdig.0000857 (PMC12068565; doi:10.1371/journal.pdig.0000857)
Supplement: Attachment — Submitted filename: Response to reviewers 1-2.pdf [file pdig.0000857.s004.pdf]

Response to Reviewer 1

1 • The title is long. I suggest simplifying it- title simplified as suggested- Line 1, 62

Abstract

2 **Background/Objectives**

• Although there is a hypothesis, the objective is not clearly stated in the abstract. Please add a clear aim for this investigation.-clarified the aim as suggested- line 26

• Also please refine the hypothesis to match the results or add briefly the impact of the three mentioned factors (comorbidity, coinfection, addiction or mental health issues) on the virtual care findings.- have matched the results to the hypothesis- line 29-31

Methods

• Please specify if the 18-item self- administered questionnaires were validated? Also, if reliability testing was performed?- this questionnaire was developed for this study and has not been validated, there was no validated tool in literature, this has been clarified in document, no reliability testing was done- line 32

• Also specify the data collection method for laboratory results.-lab results were extracted from patient records this has been clarified- line 33-34

• The terms: "COCO study" and "CD4 counts" might not be familiar for the general reader. Consider defining them.- COCO removed, CD4 clarified as CD4 T-lymphocytes- line 34

• Add a sentence specifying the statistical methods and software used.- this has been added at the request of the reviewer but is not standard reporting in an abstract- line 36-7

Results/ Key Findings

• Please add supporting statistics for the statements:" Access to and difficulty with technology was not a barrier to our older participants" and "Those with a detectable HIV viral load were more likely to engage in virtual care".- p values added-line 37-8

Conclusions

• Please avoid a generic statement in conclusion and kindly specify what recommendations study can suggest based on the specific findings.-modified as requested-line 43-5

• Please elaborate on how can virtual health policymakers or health organizations address the identified elements in the study. Example may include a targeted outreach programs to ensure all eligible patients are aware of virtual care keeping in mind the patients' preferences.-statement added as requested- line 44

Introduction

3 • Please provide reference(s)/citation(s) for the statement:"Many physician offices and hospital clinics struggled with prioritizing patients for in person assessment". Also was this specific to HIV care or healthcare in general?- reference added line 71, this was not specific to HIV and has been clarified- line 67

• Please provide reference(s)/citation(s) for the statement: "As the population of persons living with HIV in Canada and elsewhere are more economically disadvantaged, there was concern about access to virtual care"- reference and statement added- lines 74-76

• Please add stats on economic disparities among people living with HIV in Canada.- reference and associated stats added- line 74-6

• Please clarify how privacy concerns and HIV prevention measures are important in shared living spaces.- clarified the concerns around stigma and disclosure and reference added- lines 78-80

• Please provide reference(s)/citation(s) for: "With fear of contracting COVID-19 and the disruption of laboratory services, there was concern regarding delays in regular-laboratory testing with negative consequences including missed adverse drug effects, failure to detect and intervene on HIV viral failure or non-adherence and undetected sexually transmitted infections".- reference added- line 83

• Please provide the full name of all abbreviations- done

Methods

4 • Please provide the methods section after the introduction. This will help ensure clarity and smooth flow of the study.- the journal instructions were to place the methods at the end, I have moved as per the suggestion but will leave the decision to the editor- line 102

• Please specify he study design in clear statement.-study design clarified as cross sectional self reported survey- line 119

• Mention the exact period when participants completed the questionnaire. As mentioned in the abstract, data collection occurred between February 2021 and March 2023.- this added to methods- line 128

• Please clarify how participants were recruited?- clarified that the participants were approached at their planned visits to their main study visit for participation in this substudy, the number of sites of the main study who were able to participated and the proportion that consented to this substudy is in the methods- lines 113-118

• The Virtual Care Questionnaire was used. Any content validation? Any pilot testing? This is required to ensure participant comprehension before full implementation. This is important given the study population, including older adults and individuals with potential mental health or substance use challenges.- this questionnaire was developed for purposes of this study-there was no validated questionnaire in the literature- it was reviewed and approved by the community advisory board of the aging cohort and included 12 persons living and aging with HIV- lines 119-129

5 • **Results**

• "454 participants completed the questionnaire": Please provide more explanation for the recruitment process and criteria for eligible participants.- this is clarified- all participants from the sites who agreed to participate in this substudy were approached at the routine study visit to the main study- the proportion consenting is reported- there were no other eligibility criteria- lines 113-118

• Please more details on the main reasons for lack of virtual care offerings.- the details were not captured in our questionnaire

6-Discussion

• Address how study findings are relevant to global healthcare systems in similar settings.- discussed- lines 328-335

• Expand discussion on the impact of COVID-19 pandemic as the study was covering the pandemic- expanded- lines 294-6, 321-323

• Discuss how do the % of satisfaction results compare to expectations/targets/benchmarks or previous studies?- virtual care has been studied in many patient populations, our goal was to describe the experience in those with HIV and not to compare to other disease groups, but we have discussed a few reported studies- lines 301-318

• Based on the study findings, please explain in more details the reasons for higher healthcare engagement among older adults.- we have hypothesized that the older group was more concerned about their health and their comorbidities having survived with HIV- this is discussed- lines 233-236

• Please suggest what types of outreach programs are most effective in enhancing virtual care access and acceptance. Also compare in more details virtual care use vs. in-person care during the study period. You can provide a comparison table – we discussed some outreach programs- lines 323-327 - we did not capture experiences on in person care during this period as we had kept our questionnaires short, and therefore cannot compare the experiences- we have discussed this as a study limitation- line 348

Please add study limitations:

1. The study participants were actively enrolled in ongoing cohort studies and they may not reflect the broader HIV population.- added – line 345-6
2. The bias, which could influence responses regarding satisfaction and engagement in care.- added line 345

3. Findings may not be fully applicable to other healthcare settings outside of Canada.- added- line 347

• Please suggest some directions for future studies based on your study limitations and unresolved issues.- added- lines 362-369
• Also please consider the impact of changes in policies in Canada (if any).-there were no changes in policy other than the funding during COVID for virtual care, this is added- line 323
• Future studies can explore how do telehealth reimbursement models affect provider participation and patient access to virtual HIV care?- added- line 323
Recommendation for future studies- added- lines 362-369

Conclusion
• Please suggest specific strategies for improving in virtual care vulnerable communities.- done- line 370
• Please add in brief how the study results can inform policymakers or healthcare planning teams- done including work we have reported on virtual gertiatric care models in HIV- lines 370-3
• Add how can virtual care platforms better integrate multidisciplinary teams to improve holistic HIV care.- done- line 372
• Please add citation/references whenever indicated- done.

Response to Reviewer 2:

Reviewer #2: The authors present an observational cohort study that examines the impact of virtual care on vulnerable populations living with HIV in Canada during the COVID-19 pandemic. The authors surveyed participants from two established cohorts, CTN 222 (HIV/HCV coinfection) and CTN 314 (older than 65 years). They found that 55.3% of participants engaged in virtual care, with higher engagement in the older cohort. Satisfaction with virtual care was high, with 55% very satisfied, but 80% preferred in-person visits. The study highlights the need for flexible care models to meet the preferences and needs of patients.

I found the study and its findings interesting, important and presented clearly. I especially liked the clear presentation in the pie graphs, horizontal bar graphs, and colour schemes.- thank-you for your support of our work

My main concern is definition of virtual care which they simply describe as "audio or video" without further definition or details in the manuscript itself. I later found this in the Supplementary material "Virtual care is when you have your appointment with the doctor/healthcare provider over the phone or the computer". This is important because if any phone/computer/remote interaction is included in virtual care outside of an 'appointment' it would change the stats, findings, and perspective (see below).we have clarified this in the methods- line 123-25

I suggest also indicating which specific virtual services were used. For example, on the healthcare provider side, what audio and video systems/products/services were used? This could affect how often and in which ways virtual care was offered to this population (e.g. line 99-100), and 'not being offered' was a key reason for reporting non-usage of virtual care by the patients.- we did not specifically collect data on the provider or participant systems- we have added this as a limitation of the study- line 350

Similarly, on the patient side, their interpretation of what 'virtual care' means could influence their response. If virtual care does not include other times a clinician or healthcare team member provides aspects of care beyond scheduled appointments. This might especially affect whether they considered virtual care (encounters?) with allied health members such as nurses.- we framed the question as to any health care provider- in Figure 1 we report that this was most commonly with the primary care physician or HIV provider,

In Figure 1, 'not having a computer' was a reason for not participating in virtual care, but virtual care could include audio in which case only a phone may be required. Many virtual care platforms now include multi-modality communication types – video, phone/audio, and text messaging or secure messaging via patient portals or even remote patient monitoring via connected medical devices.- you are correct, however some health care providers only

delivered virtual care via video for reimbursement reasons, we outline in the discussion the need to develop multiple methods for communication- line 328-29 and provided some examples lines 334-340

My other key recommendation is to compare the virtual care usage in these cohort populations with other general and similar non-HIV populations. There are published data on general population virtual care usage in Canada over the same time periods which would inform a comparative benchmark rather than just against other HIV cohort studies. This could at least be done in the discussion if not the results.- our purpose was to describe the experience in those living with HIV and not to compare to other disease groups, but we have added some of these studies in our discussion as suggested- lines 301-318

Additional minor points:
Line 53 Introduction – states physical exams can not be done by virtual care – but inspection can often be done by phone or video, as can assessing a patients voice. Some health programs have even developed standards for guided physical exams by virtual care.- have modified the statement- line 70
Line 56 Introduction – states 'privacy of home' as a reason, but may exclude similar privacy consideration for homeless people or those at work or other locations.- clarified
Line 70 Introduction – does 'face to face' exclude video?- yes
Line 133 and the Discussion – does human contact mean in person? – yes This may depend on the type of virtual care. Other studies have reported that ongoing texting conversations have strengthened healthcare provider-patient relationships as they can be more responsive than booked appointments.
Line 158 Discussion – what virtual platform? – describe in the methods or findings- we did not specifically collect this information and have discussed that in the limitations section- line 348-50
Line 162, 208-210 Discussion – compare also to non-HIV population literature (general, and perhaps HCV/addictions and other aging disease cohorts)- as you have suggested above
Line 182 Discussion – could it be that patients with higher viral loads experience more 'virtual care' because they were called about these results or more effort was made to book/follow them up?- yes we had that in our hypothesis for this finding in our discussion- lines 246-251
Line 189 Discussion – see above re allied health and spontaneous/unscheduled phone calls-, as above , we asked about appointment in this study
Line 213 Discussion – see above about other modalities of virtual care. OK if for this paper it is clearly defined as audio and video – but perhaps the discussion can mention other types- done as suggested- lines 328-29, lines 334-340
Line 251 – Discussion – why isn't data available on in person visits? That would be helpful (though not critical)- we did not specifically collect data on in person visits during this period in order to keep the questionnaires brief, we acknowledge this in the limitations section- line 350-2

Finally, I didn't see any questions about privacy concerns from the patient survey. – this was an oversight and added to the limitations- line 352-3. This could be mentioned as a limitation or discussed. That said, privacy is not usually a concern for patients as much as virtual care administrators tend to portray it. In fact, it can improve comfort with privacy to not have to sit in an HIV clinic waiting room where they might be seen by people they know.
